# Stem Cells in the Exocrine Pancreas during Homeostasis, Injury, and Cancer

**DOI:** 10.3390/cancers13133295

**Published:** 2021-06-30

**Authors:** Sophie C. Lodestijn, Sanne M. van Neerven, Louis Vermeulen, Maarten F. Bijlsma

**Affiliations:** 1 Laboratory for Experimental Oncology and Radiobiology, Center for Experimental and Molecular Medicine, Cancer Center Amsterdam and Amsterdam Gastroenterology Endocrinology and Metabolism, Amsterdam University Medical Centers, Meibergdreef 9, 1105 AZ Amsterdam, The Netherlands; s.c.lodestijn@amsterdamumc.nl (S.C.L.); s.m.vanneerven@amsterdamumc.nl (S.M.v.N.); l.vermeulen@amsterdamumc.nl (L.V.); 2 Oncode Institute, Meibergdreef 9, 1105 AZ Amsterdam, The Netherlands

**Keywords:** pancreas, pancreatitis, pancreatic cancer, pancreatic stem cells, stem cell dynamics, microenvironment

## Abstract

**Simple Summary:**

Pancreatic cancer is one of the most lethal malignancies. Hence, improved therapies are urgently needed. Recent research indicates that pancreatic cancers depend on cancer stem cells (CSCs) for tumor expansion, metastasis, and therapy resistance. However, the exact functionality of pancreatic CSCs is still unclear. CSCs have much in common with normal pancreatic stem cells that have been better, albeit still incompletely, characterized. In this literature review, we address how pancreatic stem cells influence growth, homeostasis, regeneration, and cancer. Furthermore, we outline which intrinsic and extrinsic factors regulate stem cell functionality during these different processes to explore potential novel targets for treating pancreatic cancer.

**Abstract:**

Cell generation and renewal are essential processes to develop, maintain, and regenerate tissues. New cells can be generated from immature cell types, such as stem-like cells, or originate from more differentiated pre-existing cells that self-renew or transdifferentiate. The adult pancreas is a dormant organ with limited regeneration capacity, which complicates studying these processes. As a result, there is still discussion about the existence of stem cells in the adult pancreas. Interestingly, in contrast to the classical stem cell concept, stem cell properties seem to be plastic, and, in circumstances of injury, differentiated cells can revert back to a more immature cellular state. Importantly, deregulation of the balance between cellular proliferation and differentiation can lead to disease initiation, in particular to cancer formation. Pancreatic ductal adenocarcinoma (PDAC) is a lethal disease with a 5-year survival rate of only ~9%. Unfortunately, metastasis formation often occurs prior to diagnosis, and most tumors are resistant to current treatment strategies. It has been proposed that a specific subpopulation of cells, i.e., cancer stem cells (CSCs), are responsible for tumor expansion, metastasis formation, and therapy resistance. Understanding the underlying mechanisms of pancreatic stem cells during homeostasis and injury might lead to new insights to understand the role of CSCs in PDAC. Therefore, in this review, we present an overview of the current literature regarding the stem cell dynamics in the pancreas during health and disease. Furthermore, we highlight the influence of the tumor microenvironment on the growth behavior of PDAC.

## 1. Introduction

**Figure 1:**
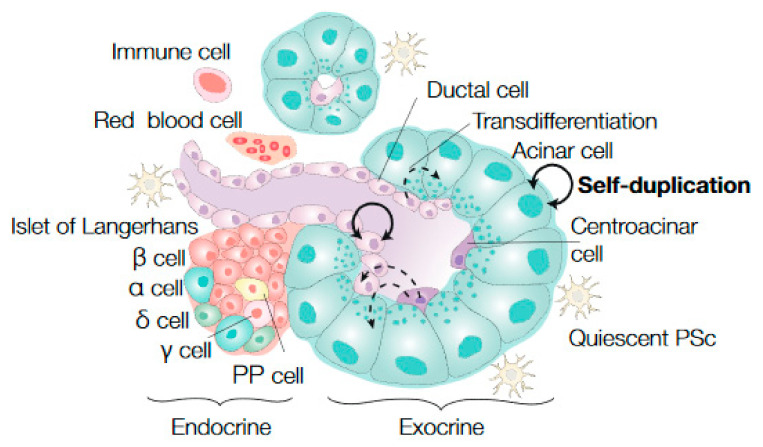
Stem cell dynamics in the exocrine pancreas during homeostasis. The pancreas consists of an endocrine and an exocrine compartment. The endocrine cells are clustered in islets (the islets of Langerhans) and include α cells, β cells, δ cells, pancreatic polypeptide (PP), and γ cells. The exocrine compartment contains enzyme-producing acinar cells, a ductal system that is lined up with ductal cells and centroacinar cells, which are located at the tips of ducts, between the acinar and ductal cells. Important microenvironmental factors of the homeostatic pancreas are immune, red blood, and quiescent pancreatic stellate cells (PSc). The current literature on stem or progenitor cells in the exocrine homeostatic pancreas predominantly points to a model in which homeostasis is maintained by self-renewal of mature pancreatic cell types (self-duplication, solid black arrows). However, few (lineage tracing) studies identified multipotent progenitor cells (ductal and centroacinar cells) that were able to transdifferentiate to other exocrine pancreatic cells (acinar and ductal cells), albeit rare events (indicated by dashed arrows).

The pancreas is a metabolic organ that consists of two main parts: the endocrine and exocrine compartment. The endocrine compartment regulates the blood glucose levels by distinct endocrine cell types that are clustered in islets (the islets of Langerhans), including α cells, β cells, δ cells, and pancreatic polypeptide (PP) or γ cells, which secrete hormones, including glucagon, insulin, somatostatin, and pancreatic polypeptide, respectively. The exocrine compartment contributes to food digestion, which is facilitated by two major cell types; the acinar cells, which produce enzymes that are transported to the gut via a ductal system that is lined up with the other prominent cell type, and ductal cells [1,2]. A third exocrine cell type is the centroacinar cells, which are physically located at the tips of ducts between the ductal and acinar cells. Their function is not completely understood [2] (Figure 1).

Diseases of the pancreas arise when one of these compartments is deregulated. For instance, severe metabolic conditions, such as diabetes mellitus, emerge when the endocrine β cells stop producing insulin (diabetes type 1) or the peripheral tissue becomes insensitive for the secreted insulin (diabetes type 2) [1,3,4,5]. Deregulation of the cellular organization of the exocrine compartment can result in the formation of the most common form of pancreatic cancer; pancreatic ductal adenocarcinoma (PDAC). An important risk factor to develop PDAC is chronic pancreatitis [6,7,8,9]. PDAC is one of the most lethal diseases, with an overall survival rate of less than 10% at 5 years from diagnosis [10]. This dismal prognosis can, in part, be attributed to late detection, as ~85% of all PDAC patients present with non-resectable, advanced disease and metastases [10]. Moreover, PDAC tumors exhibit a high degree of heterogeneity, which makes them resistant to most conventional radio- and chemo-therapeutic strategies. This resistance and the resulting poor prognosis of PDAC patients underscores the urgent need for a better understanding of the biology of the pancreas in health and disease. In particular, comprehending the mechanisms of therapy resistance will greatly aid the development of improved therapies for PDAC.

The principles of therapy resistance have been under debate for years, and several theories have emerged to explain this resistance. Naturally, the acquisition of genetic mutations that confer resistance to therapy have been well-described [11,12]. Additionally, the composition of the tumor microenvironment has been described to explain therapy resistance. In particular, the pervasive growth of dense fibrous tissue within PDAC tumors could create a physical barrier that prevents chemotherapy to kill tumor cells [13]. In addition, it has been suggested that resistance can be caused by a subpopulation of stem-like cells, referred to as cancer stem cells (CSCs) [14,15]. According to this theory, a tumor can be considered an aberrant organ that arose from a cell that acquired stem-like characteristics. These CSCs, like their healthy counterparts, possess the ability to self-renew while simultaneously forming a variety of cell types with diverse functions [16]. In this respect, therapy resistance is explained by the failure of conventional chemo- and radiotherapies to eradicate the CSCs, which subsequently reconstitute the tumor and results in disease relapse.

However, emerging evidence, also within the field of pancreas biology, demonstrates that cellular states are not a static but rather a plastic entity and that these states can be influenced by intrinsic and extrinsic factors throughout life [17]. For example, differentiated, mature pancreatic cell types can dedifferentiate to a more progenitor or stem-like cell state. Furthermore, in the case of severe tissue perturbation, dedifferentiated and mature pancreatic cells can give rise to other pancreatic cell types. This type of conversion is referred to as transdifferentiation [17,18]. With this in mind, therapeutic intervention could drive cancer cells towards a cellular state that no longer depends on the drug-targeted pathway.

Irrespective of the theory, it becomes evident that therapy resistance is caused by a cell population that is evolutionary ‘fitter’ or better adapted than the rest of the tumor bulk. To identify and potentially target this population, it is important to understand the biogenesis and homeostasis of a healthy pancreas, as tumor cells often hijack the organ’s own regulatory mechanisms. Therefore, the most elemental question to be addressed is whether stem cells are present in the adult pancreas.

By definition, multipotent or stem cells are characterized by their ability to generate numerous different cell lineages and their capability of long-term self-renewal [19]. Bona fide stem cells are found in many organs, including the hematopoietic system [20], mammary tissue [21,22], the skin [23,24,25] and the intestine [26]. Distinct stem cell properties can be evaluated using various experimental strategies. However, there is still a debate regarding the existence and contributions of multipotent progenitor or stem cells in the adult pancreas during homeostasis, regeneration following injury, and pancreatic cancer [27,28,29].

### Strategies to Evaluate Stem Cell Qualities

To investigate stem cell function, several assays are available. In recent years, lineage tracing experiments have proven to be very useful to study stem cell properties in different tissue types. In lineage tracing assays, a potential stem cell population is genetically marked, usually following permanent activation of a reporter in cells that express a defined stem cell marker gene. The offspring of these cells will also express this reporter and can thereby be followed over time. The advantage of lineage tracing is the ability to analyze properties of cells in their native environment. A disadvantage is that only the behavior of the cells expressing the marker gene can be studied, and other cell populations remain untested. Other strategies to identify stem cells are transplantation assays in animals and colony formation assays in vitro. With these assays, the clonogenic capacity of putative stem cells is examined by the ability to contribute to tissue (or tumors) initiation in animals or colonies in vitro, respectively. Using transplantation assays, multipotency of putative stem cells is tested by the capacity to form different cell lineages in organs or cancer tissue or in cell culture systems. A disadvantage of transplantation assays is the disruption of the original tissue organization and micro-environment. This is particularly important as it has become well established that micro-environmental cues define stem cell properties in situ [30,31].

It is important to recognize that different features of stem cells require dedicated assays to evaluate. For example, stem cell phenotype can be evaluated by studying stem cell marker expression or morphological features. However, stem cell identity, for example, by expression of tissue specific stem cell markers, does not necessarily imply stem cell function. Instead, stem cell activity, an intrinsic stem cell property, defined by multipotency and self-renewal capacity, can be functionally tested using lineage tracing experiments. Simultaneously, stem cell potential, defined as the potential of non-stem cells to re-obtain stem cell activity, can, for instance, be evaluated using lineage tracing after injury induction of ablation of specific cell populations. Finally, stem cell functionality, refers to a feature of the stem cell pool as a whole, more specifically, the cells that contribute to tissue renewal and can be defined using clonal dynamics assays in combination with mathematical modelling (reviewed in ref. [32]).

A better understanding of the stem cell dynamics of the healthy and injured pancreas will aid the development and improvement of treatment strategies for pancreatic diseases, such as diabetes and PDAC. Most literature reviews regarding stem cell dynamics in the pancreas are focused on the endocrine compartment to explore possibilities to repopulate the exhausted pool of β cells by stem or progenitor cells in patients with diabetes. In this review, we outline the evidence for the existence and functionality of stem cells in the exocrine pancreas during homeostasis, injury, and pancreatic cancer to create an overview for potential therapy options to treat patients with PDAC. In addition, we will review the current literature on cellular plasticity of the pancreatic tissue in different circumstances and the role of the micro-environment in regulation stem cell functionality.

## 2. Stem Cell Dynamics in Healthy Pancreatic Tissue

The pancreas starts to develop from the endoderm of the embryonic foregut from 8.5 days of mouse gestation (E8.5), comparable with about 3 weeks in human embryos [33]. The pancreas originates from fusion of two pancreatic buds in the endoderm at E11.5, and all main cell types are present at E15.5 [34]. Studies have shown that the most prominent pancreatic cell lineages, exocrine and endocrine cells, arise from the same pool of multipotent progenitor cells during embryogenesis [35,36]. Early on during development, these multipotent progenitor cells express the transcription factor Pancreatic and Duodenal Homeobox 1 (*Pdx1*) and can give rise to all pancreatic cell lineages. *Pdx1*^+^ progenitor cells are generated until approximately E12, and after this period, regeneration of the pancreas does not occur when progenitor cells are removed [37].

After this early developmental phase (around ~E12), the pancreas becomes more compartmentalized into a “tip” and “trunk” domain, and this process results in lineage restriction of both compartments. The tip domain consists of acinar cell fate progenitors and the trunk domain of progenitor cells with predominant endocrine and ductal differentiation potential [36,38]. Pancreas transcription factor 1 complex (*Ptf1a*) and carboxypeptidose A1 (*Cpa1*) expression become restricted to the tip domain, whereas expression of markers as *Sox9*, *Hnf1b*, and *Nkx6.1* are exclusively expressed in the trunk domain [36,38,39,40,41]. Only recently, by using clonal fate mapping, it was established that, also during late development (after ~E12.5), heterogeneity within cell lineage potential exists [42,43]. Multipotent- and acinar-restricted progenitor cells, located at the tips of growing ducts, drive the formation of the pancreas. This occurs in a stochastic process of ductal branching, in which the branching process terminates by signals from a neighboring expanding branch [43]. Clonal tracing after this late period of pancreatic development showed even more lineage restriction with the existence of only very sparse bipotent ductal-acinar clones after E18.5 [43].

Single cell RNA sequencing efforts have increased our understanding of pancreatic lineage specifications during embryogenesis [44]. These studies mainly identified cellular heterogeneity in the pool of endocrine embryonic progenitor cells [45,46,47]. Recently, transcriptomic analyses of pancreatic cells from different genetically engineered mouse models during development (E9.5–E17.5) revealed a comprehensive overview of the embryonic cell lineage trajectories that captures and partly confirms the data described above [48]. Different multipotent progenitor populations were identified, whereby early progenitor cells (E9.5) expressed *Pdx1* and late progenitor cells (E10.5–E11.5) expressed *Ptf1a* and *Sox9*. The late progenitor cells subsequently developed into tip cells, which then branched into acinar and trunk cells. The early trunk cells are considered bi-potent and generate either ductal cells or endocrine progenitor cells, which differentiate into mature endocrine cells via intermediate progenitor cells stages [48]. Although these transcription data-based insights can help to identify possible markers for pancreatic stem cells, functional experiments are necessary to validate these findings.

The extensive proliferation and expansion of developing pancreatic tissue allows for clonal tracing during this period. This is in contrast to the adult pancreatic tissue, which is rather dormant, as reflected by its proliferation rates: less than 2% of the cells proliferate in the adult pancreas compared to ~40% directly postnatally [49]. As a consequence, our understanding of the clonal dynamics and the exact process of renewal of the exocrine cells during homeostasis in the adult pancreas is limited, as assays that would assess that this takes a lot of time [2,29]. In particular, it remains unclear whether a specific subpopulation of the exocrine pancreas has the ability to generate new cells, and thereby functions as stem-like cells in the adult pancreas, or if all mature cells have these properties. Despite the low turnover rate in the adult pancreas, few lineage tracing studies have been performed from mature acinar cells postnatally and during homeostasis [49,50,51,52,53]. In these studies, it was demonstrated that cell lineages become restricted and that self-duplication is the main mechanism of tissue development postnatally (Figure 1). However, within the mature acinar population *(Bmi1*^+^ and *Nestin*^+^ cells), variation in the capacity for clonal expansion and prolonged self-renewal was observed [50,51]. On the other hand, an older study demonstrated that cells expressing the terminal acinar differentiation marker *Elastase* were also capable of successful regeneration [52], which implies that all acinar cells have some degree of self-renewal and progenitor capacity.

In addition to the evidence for unipotency in the adult acinar cells, multiple studies showed similar results for the ductal lineage [38,41,54]. By following *Hnf1β*-expressing cells during development and post birth, it was demonstrated that these cells become more lineage-restricted during development, resulting in an unipotent cell population that only generates ductal cells postnatally [38]. Additionally, Kopinke et al. showed that the Notch-responsive *Hes1*-positive ductal and centroacinar cells are unipotent and do not contribute to β-cell formation in the adult pancreas [54]. In agreement, *Sox9*-expressing cells were found to predominantly generate ductal cells in the normal adult pancreas, while they were able to give rise to endocrine and acinar cells during development [41] (Figure 1). In notable contrast, lineage tracing data, obtained from *Sox9*-expressing cells under physiological conditions in adult mice, suggested that these cells function as exocrine progenitor cells that can become ductal, centroacinar, and acinar cells but not islet cells [55]. Recently, using in vitro and in vivo experiments, a subpopulation of centroacinar cells with multipotent capacities was identified. This subpopulation expresses *Aldh1b1*, and single cell RNA-sequencing provided its molecular signature and suggested the preferential expression of *Kras* in this subpopulation [56]. As such, this population has been proposed as the cell of origin for PDAC.

Interpretation of these and other studies is complicated because of several technical caveats. For example, in vivo lineage tracing studies are difficult to compare and interpret due to the use of different cell type specific promoters, inducible systems, and dosage of promoter inducers. Importantly, the inducer itself can also influence the clonal dynamics. For instance, it has been reported that tamoxifen induces cell death in stomach and intestinal tissues [57,58] and proliferation in the acinar cell population [59]. As a result, tissue regeneration is tested rather than homeostasis. Furthermore, lineage tracing studies are fundamentally biased by studying only one cell population and thereby leaving unmarked cell populations untested.

Furthermore, in vitro clonogenic assays, colony formation assays with the ability to form organoids, and transplantation assays showed evidence for the existence of subpopulations of exocrine pancreatic cells with stem or progenitor features, including ALDH1^+^ cells (centroacinar and terminal ductal) and Nestin^+^ cells (islet and ductal) [60,61,62,63,64,65,66]. The in vitro studies and transplantation assays described above suggest the existence of stem cells in the adult pancreas. However, these experiments test stem cell features in a non-physiological way by disrupting the original tissue organization and using artificial culture conditions

In summary, the current data on stem cell dynamics in the homeostatic pancreas predominantly points to a model in which homeostasis is maintained by self-renewal of mature pancreatic cell type, and multipotent progenitors, dedifferentiation, and transdifferentiation are rare events.

## 3. Pancreatic Stem Cell Dynamics during Regeneration

In contrast to the homeostatic pancreas, the proliferation rate is high during tissue regeneration after injury [67,68]. However, it is still unclear how this upregulated proliferation is regulated. For example, it is unknown to what extent stem or progenitor cells play a role in this regeneration process or whether cellular plasticity is mainly responsible for tissue repair. Previously, a hybrid model, the facultative stem cell model, has been proposed to describe a situation in which tissues consist of unipotent cells that have the capacity to function as stem cells upon tissue damage [69]. To elucidate which cells contribute to tissue regeneration of the pancreas, several different models of pancreatic injury were employed, such as chemical-induced pancreatitis, partial duct ligation, partial pancreatectomy, and β-cell ablation.

### 3.1. Ductal Cells as Progenitor Cells during Regeneration

**Figure 2:**
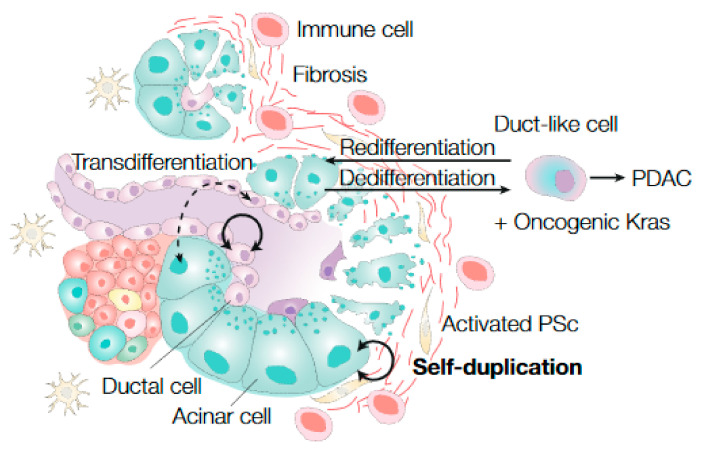
Stem cell dynamics in the exocrine pancreas during injury. After injury induction, the pancreas becomes fibrotic, immune cells infiltrate, pancreatic stellate cells (PSc) become activated, and the cellular differentiation state of exocrine pancreatic cells (ductal and (centro) acinar cells) becomes more plastic. Cellular replacement of pre-existing exocrine cells (self-duplication), often via dedifferentiation to a more immature state (duct-like cell), is the most important mechanism for pancreatic tissue regeneration upon injury (solid black arrows). Infrequently, after persistent or severe inflammation, mature exocrine pancreatic cells can transdifferentiate into other pancreatic cell types (acinar to ductal cells and vice versa; dashed black arrow). Injury in combination with oncogenic *Kras* induction in acinar cells can ultimately give rise to initiation of pancreatic ductal adenocarcinoma (PDAC), mostly via pancreatic intraepithelial neoplasia (PanIN).

Based on the finding that the ductal cell population can act as a multipotent progenitor cell during early development, it was speculated that these cells might also function as progenitor cells following injury [38,41,70]. This hypothesis was further supported by the observation that embryonic progenitor markers, such as *Pdx1*, *Notch*, and *Ngn3*, were re-expressed after injury [71,72,73,74]. Similar results were observed for *Lgr5* expression, a Wnt agonistic receptor. In the homeostatic pancreas, Wnt signalling is inactive [75], while it is important for its development during embryogenesis [76,77]. After partial duct ligation, the Wnt signaling pathway is reactivated, concomitant with the appearance of *Lgr5* expression in ductal cells [64]. Based on direct lineage tracing from the ductal cell compartment, most studies found no evidence for ductal cells as progenitor cells following injury [38,41,54,55,78] (Figure 2). However, others described that ductal cells expressing *Ngn3* or *CAII* are able to differentiate towards endocrine or acinar cell lineages upon damage [72,74,79,80,81,82]. These partially conflicting results might be explained by inducing different degrees of injury with different injury models [74]. Since embryonic progenitor markers as *Pdx1* and *Ngn3* were re-expressed during this cell conversion, it was assumed that this process occurred via dedifferentiation [74]. Interestingly, Sancho et al. demonstrated that loss of a single gene, *Fbw7*, stabilizes *Ngn3*, the key embryonic regulator of endocrine cell fate, and results in reprogramming CK19^+^ ductal cells to an α-, β-, and δ-cell type [83].

Other than the questions regarding the functionality of multipotent ductal cells, little is known about their exact location or niche within the injured pancreas. It has been shown that putative endocrine and ductal progenitor cells are located in close proximity to ductal structures [72,84]. Additionally, pancreatic ductal glands, comprised of ductal cells, expressing embryonic progenitor markers as *Sox2* and *Nanog*, were suggested to serve as an important progenitor niche for tissue repair [85].

As outlined, the data on whether ductal progenitor cells exist in the injured pancreas are not fully concordant. This could be the result of the different promotors used for lineage tracing and the dissimilar injury models used to study ductal cell fate. Overall, most lineage tracing studies showed results that argue against the presence of a ductal progenitor cell for acinar and endocrine generation upon tissue damage, but based on the current data, this cannot be entirely ruled out, and more specific research is necessary to clarify the different observations.

### 3.2. Acinar Cell Plasticity during Regeneration

In case of injury, acinar cells morphologically change to duct-like structures and dedifferentiate by re-expressing developmental progenitor markers, including *Pdx1*, *Hes1*, *Sox9*, and *Hnf1β*. At the same time, the expression of mature acinar cell markers (e.g., amylase) decreases [73,86,87,88]. For this reason, the term “duct-like cells” has been used to describe these dedifferentiated acinar cells, which have also been suggested to act as stem cells [17,86]. Indeed, most studies showed evidence for a regeneration process in which dedifferentiated acinar cells restore the exocrine compartment upon tissue injury [51,52,53,73] (Figure 2). Notably, this regeneration process of acinar cells is regulated by interaction with immune cells that are present as a result of the inflammatory response after injury induction [89]. Lineage tracing from an acinar specific promotor in combination with cerulein-induced pancreatitis did not show evidence for transdifferentiation of acinar cells into other pancreatic cell types [51,53,73]. Likewise, other injury models, such as pancreatectomy and partial duct ligation, also demonstrated no conversion of the acinar cell type to ductal or endocrine lineages [52] (Figure 2). Doublecortin-like kinase-1 (*Dclk1*) has been shown to be an essential gene for proper regeneration upon acute and chronic cerulein-induced pancreatitis, as *Dclk1*^+^ cells started to proliferate rapidly after injury to repopulate the acinar cell compartment [90]. During homeostasis, *Dclk1* is predominantly expressed by acinar cells, although rare ductal cells express it as well, and this marks a quiescent and long-lived cell population [90]. These results highlight the existence of heterogeneity in regeneration capacity within the acinar cell compartment to regenerate the pancreas after tissue damage.

While these findings propose that self-renewal of pre-existing acinar cells is the main mechanism of regeneration upon injury, other reports argue that acinar cells can transdifferentiate to recover from pancreatitis [88,91,92]. Of note, in these studies, other methods and mouse models were used to investigate the multipotent potential of acinar cells during inflammation. For instance, adenoviral delivery in acinar cells of three important β-cell transcription factors, *Ngn3*, *Mafa*, and *Pdx1*, resulted in a phenotype that closely resembled β-cells in vivo [91]. Further evidence for multipotency of acinar cells during injury was obtained by performing lineage tracing. In these studies, it was observed that acinar cells transdifferentiated to ductal cells [92] and endocrine cells [88] to repopulate damaged tissue. Interestingly, the transition from acinar to β-cells occurred via endocrine (*Ngn3^+^)* and ductal (*Ck19^+^/Hnf1β^+^/Sox9^+^*) progenitor cells [88]. Only partial duct ligation was sufficient to induce this cellular transformation process; however, it could be enhanced by the addition of streptozotocin treatment to selectively eliminate pre-existing β-cells [88]. This suggests that, similar to ductal cell plasticity, the degree of injury might be crucial in defining multipotent capacity of acinar cells following injury [52]. Despite this, the high labeling efficiency and analysis of the tissue after a very long period of injury by Pan et al. could explain the conflicting findings.

In conclusion, the cellular state of the acinar cell type seems to be plastic during and after inflammation of the pancreas, based on studies that showed re-expression of embryonic markers in differentiated acinar cells upon injury. Cellular replacement of pre-existing acinar cells, either direct or indirect via a dedifferentiated state, seems to be the most important mechanism for pancreatic tissue regeneration upon damage. Nevertheless, upon severe injury, some mature acinar cells seem to have the ability to transdifferentiate into other pancreatic cell types, either directly or via an intermediate progenitor state or other cell type. For future studies, it will be important to provide quantitative analyses of these phenomena to determine the relative contributions of the various regenerative programs.

## 4. Pancreatic Stem Cell Dynamics during Cancer Initiation and Cancer

As described above, cell fates of mature pancreatic cells can become plastic under pathological conditions. When inflammation of the tissue occurs, differentiated pancreatic cells are capable of dedifferentiation to a stem-like condition, or even directly transdifferentiate to another cell type. Generally, when the injury is short-term or not sufficiently severe, dedifferentiated cells will revert back to their original differentiated cell fate to repopulate the damaged tissue. Conversely, if the injury is maintained or very severe, for instance during chronic pancreatitis, oncogene activation can occur and dedifferentiated cells might lose cell fate constraints [17]. The notion that this mechanism can ultimately lead to the development of pancreatic cancer is supported by multiple studies (reviewed in ref. [93]).

### 4.1. The Cell of Origin in Pancreatic Cancer

Pancreatic cancer arises from neoplastic precursor lesions, mostly from pancreatic intraepithelial neoplasia (PanIN) and more sporadically from intraductal papillary mucinous neoplasia (IPMN) or mucinous cystic neoplasm (MCN) [11,94]. During this transformation of neoplastic precursor lesions to PDAC, several mutations are often observed, including activation of *KRAS* oncogene, inactivation of the tumor-suppressor gene *CDKN2A,* and inactivation of the tumor suppressor genes *TP53* and *SMAD4*, being present in approximately 90%, 95%, 50–75%, and 55% of the tumors, respectively [11,12]. These mutations have been functionally confirmed to contribute to PDAC formation by using genetic mouse models in which activation of *Kras* in combination with *Trp53* or *Cdkn2a* resulted in the formation of metastatic PDAC [95,96,97]. However, the question of which pancreatic cell type functions as the cell of origin for these precursor lesions and PDAC remains inconclusive, and data to support both the ductal and the acinar cell as potential cells of origin have been described [86,96,98,99,100,101,102,103,104].

The expression of ductal markers, such as CK19, and the histological ductal morphology of PDAC led to the assumption that PDACs arise from the ductal cells [93]. However, in transgenic mice, *Kras* activation in ductal cells expressing CK19 did not result in PDAC but instead resulted in pancreatic periductal lymphocytic infiltration and gastric mucous neck cell hyperplasia [103]. Other research showed that deletion of the Brahma-related gene 1 (*Brg1*), part of the chromatin remodeling SWI/SNF complexes in combination with Kras activation, promotes PDAC via IPMN formation in adult ductal cells, suggesting that the ductal cell is indeed the cell of origin for the small subset of IPMN-PDACs. Interestingly, during transformation, ductal cells first dedifferentiated, as confirmed by expression of progenitor markers, such as *Pdx1* and *Hnf4a* [104]. In addition, the function of the Brg1 protein during this transformation is context-dependent, as it has a tumor suppressive function in the early phase by inhibiting dedifferentiation of the ductal cells but stimulates tumor growth within established tumors by inducing epithelial to mesenchymal transition [104].

Most of the evidence currently supports acinar cells as initiators of development of PanIN and PDAC. Lineage tracing from different acinar specific promotors in a *Kras*-driven mouse model is frequently used to investigate acinar cell transformation and demonstrates that *Kras* activation in the acinar compartment is sufficient to induce PanIN lesions that can ultimately progress to PDAC [86,96,98,99,100,101,105,106]. In addition, the susceptibility of the acinar cells to this *Kras*-driven oncogenic transformation seems to differ between different life stages. While the acinar cells are more susceptible for this transformation during embryogenesis and early development, the adult acinar cells seem to be refractory to *Kras*-induced PanIN and PDAC development [96]. Interestingly, combining *Kras* activation with the induction of mild chronic inflammation enhances oncogenic transformation in these adult acinar cells [96]. Notably, β-catenin activity appears to restrict acinar-derived *Kras*-induced formation of PanINs, while β-catenin signaling is activated during regeneration of acinar cells upon acute pancreatitis induction and is known to regulate acinar cell development during embryogenesis [86]. However, a minimal threshold of Wnt signaling seems to be required for PanIN formation [107]. The opposite is observed for the Hedgehog pathway, which is absent during development and homeostasis and increases during progression from PanIN stages to carcinoma formation [108,109]. Indeed, the Hedgehog pathway plays an important role in PDAC formation and progression, both in pre-clinical PDAC models [110,111] and in human PDAC [108,112]. These results suggest that embryonic signaling pathways can be differentially regulated during embryogenesis, regeneration after injury, and development of neoplastic precursor lesions.

Despite a different temporal susceptibility for oncogenic transformation of acinar cells, susceptibility differences within the acinar cell population are observed [90,105,113]. For instance, acinar cells expressing *Dclk1*, a microtubule regulator, are quiescent in combination with Kras activation but initiate cancer upon cerulein-induced pancreatitis [90]. These results imply that a subpopulation of acinar cells in conjunction with an activated oncogene is more prone to initiate pancreatic cancer. Additionally, while *Sox9* expressing ductal and centroacinar cells seems to be refractory to *Kras*-driven oncogenic formation, concomitant expression of *Sox9* and *Kras* accelerates the formation of acinar-derived PanIN lesions [105]. This indicates that neoplastic precursor lesions arise from acinar cells via the induction of a duct-like or a dedifferentiated state [105], as was also observed in prior studies [86,96] (Figure 2).

In conclusion, current evidence favors the acinar cells as the cell of origin for PDAC development via PanIN lesions, whereby dedifferentiation occurs prior to acinar-to-ductal metaplasia. The susceptibility of the pancreatic cells for this oncogenic transformation is dependent on different factors, including oncogenic activation, environmental factors, and timing. PDAC tumors derived from IPMN lesions are likely to arise from the ductal cells via a similar dedifferentiation process. This suggests that the cellular source of PDAC might determine the transformation process.

### 4.2. Stem Cell Identification and Functionality in PanIN, PDAC, and Metastasis

**Figure 3:**
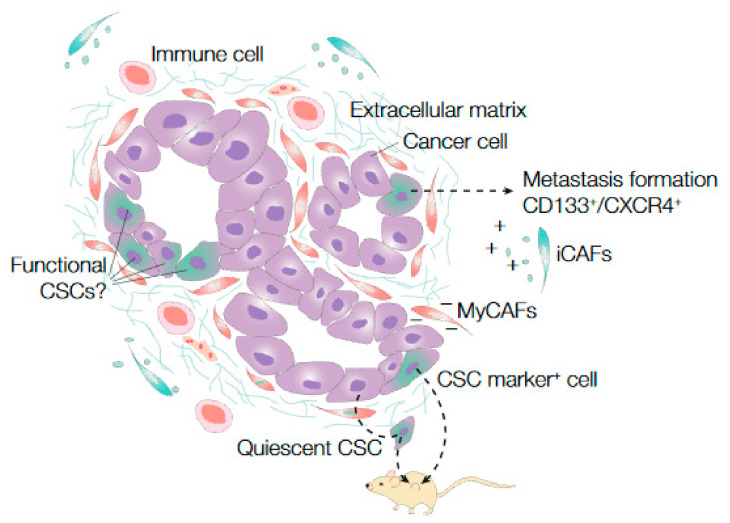
Cancer stem cell dynamics in PDAC. Pancreatic ductal adenocarcinoma (PDAC) is characterized by thick stromal fibers (extracellular matrix) that compress blood vessels and cause hypoxia. In PDAC, different putative cancer stem cell (CSC) markers have been identified in cancer cell populations that showed self-renewal, multipotency, or metastasis formation (CD133^+^/CXCR4^+^ cells) capacity after transplantation in immune compromised mice. Serial lineage tracing experiments showed that quiescent CSCs become activated after transplantation in mice. Little is known about the cancer cells that drive tumor progression (the functional CSCs) within established PDAC tissue. Different microenvironmental factors, including subpopulations of fibroblasts that secrete tumor growth promoting factors (iCAFs) and immune cells, are suggested to influence CSC functionality in PDAC. Meanwhile, other CAF subpopulations, such as myCAFs, are proposed to be tumor growth restrictive.

As described earlier, CSC properties can be tested with various assays. Using these assays, many pancreatic CSC markers have been discovered, including, CD133 [14,114,115,116], CD24 [15,117,118], CD44 [15,117,119,120], ESA [15], Nestin [121], and *Dclk1* [90,113,122] or a combination of these markers [14,15] (Figure 3). In addition, it has been shown that migration of CD133^+^/CXCR4^+^ cancer cells is essential in metastasis formation of human pancreatic cancer [14] (Figure 3).

By using unique genetic labeling of all PDAC cells in serial xenotransplantation assays, it was recently shown that tumor initiation and long-term tumor progression is driven by transiently active cancer cells [123]. These results imply that quiescent cancer cells can become active upon re-transplantation (Figure 3). Of note, in this study, CSC functionality during long-term tumor progression was tested by assays in which the tumor cells were dissociated. Therefore, conclusions about CSC functionality during tumor progression within an established tumor cannot be drawn based on these results.

Lineage tracing has also previously been used to demonstrate that metastases of PDAC are often formed by seeding of different subclones derived from the primary tumor and occur late during cancer formation [124,125]. Strikingly, the metastatic site determines the subclonal outgrowth, resulting in either monoclonal outgrowth in the liver and lung or polyclonal outgrowth in the peritoneum and diaphragm [124]. These and other results highlight the major impact of the microenvironment on the cellular dynamics; therefore, understanding the role of microenvironmental factors on stem cell functionality in PDAC is very important.

### 4.3. Microenvironmental Impact on Stem Cell Functionality in PDAC

Our views on CSCs are changing. Previously, CSCs were considered to be a subpopulation of cancer cells that exhibited intrinsic stem cell properties, such as self-renewal and the capacity to give rise to different cell lineages in a unidirectional way. However, evidence is emerging that the stem cell properties in solid tumors are plastic and that the microenvironment defines the stem cell functionality [126,127]. A better understanding of this interplay between the microenvironment and the CSCs in PDAC could help to identify potential new targets for therapy development.

The microenvironment of pancreatic tumors is abundantly present and is composed of several tumor-extrinsic factors, including immune cells, nerves, blood vessels, the lymphatic system, fibroblasts, and extracellular matrix components (e.g., fibronectin, collagen, and hyaluronic acid) [13,128]. The formation of very dense collagen fibers, described as the desmoplastic reaction, is characteristic for PDAC [129,130]. As mentioned earlier, these dense stromal fibers are assumed to increase the interstitial pressure, causing blood vessel compression and reduced drug and oxygen delivery [130]. Several studies attempted to target the stroma to enhance drug delivery and thereby improve PDAC therapy. Although this strategy showed promising results in a mouse model [110], it failed to demonstrate a positive effect on survival of patients with pancreatic cancer (reviewed in [131]). It is important to consider, in this respect, the known dichotomous role of the stroma in PDAC; both tumor-promoting [132,133,134,135] and tumor restraining [136,137,138,139] contributions have been described.

Subsequent preclinical work demonstrated that the negative effect on tumor growth by targeting the stroma in established tumors could be caused by an altered CSC population [137,138,140,141,142] (Figure 3). Additionally, other factors of the microenvironment, such as immune cells, seem to promote tumorigenicity and stemness in PDAC [143,144] (Figure 3). More precise specification of the contributions of different tumor infiltrating immune cells on the clonogenicity of cancer cells might lead to novel therapy targets for PDAC. In addition, profiling the tumor immunophenotype could potentially serve as a tool to predict therapy response of metastatic PDAC [145,146]. Notwithstanding, tumor cell biomarkers that associate with stemness have been identified, such as miRNAs, suggesting that cell-intrinsic properties contribute to clonogenicity and therapy resistance [145].

Failing of treatment by targeting the stroma might also be explained by the heterogeneity within the stromal CAF population. Recent efforts to delineate the CAF populations provided important insights. Myofibroblast-like CAFs (myCAFs), which are mainly located in close proximity to cancer cells, are assumed to function as mechanical barriers that prevent cancer cell dissemination and tumor growth, whereas, another CAF population, the inflammatory subtype (iCAFs), located further away from the tumor cells, secretes factors such as IL-6, which are assumed to promote tumorigenicity [138,147] (Figure 3). Interestingly, inhibition of the stroma by administration of a Hedgehog pathway inhibitor differently affects these CAF populations in PDAC mouse tumors by reducing myCAF numbers and increasing iCAF numbers [148]. Furthermore, another CAF subtype, with features of both iCAFs and myCAFs, has been described, suggesting the possibility of interconversibility between CAF subtypes [149].

The contribution of both CSC and stromal heterogeneity elaborates the need to understand their interplay and improve current treatment strategies for PDAC. Combining therapies to target the stroma and cancer cells might be more effective than targeting a single aspect of the tumor. Several studies showed positive results of combining therapies to target the stroma, as well as tumor cells [150,151]. However, future studies need to be performed to identify the optimal targets of different tumor compartments.

## 5. Conclusions

Compelling evidence for a specific stem cell population in the homeostatic pancreas is lacking. Instead, homeostasis and regeneration in the pancreas appear to rely on repopulation by existing progenitor cells with an equal contribution potential. Interestingly, after inducing severe damage to the pancreas, mature acinar cells dedifferentiate before repopulating the damaged tissue. This plasticity between differentiated and undifferentiated cells is not only observed during severe pancreatitis but has also been described for cancer cells. Indeed, the cellular hierarchy existing within established tumors appears to be a very plastic process in contrast to a static progress, which was previously assumed. In this process of differentiation and dedifferentiation, the micro-environment plays an important role. Several micro-environmental factors have been identified that influence clonogenicity, both derived from mesenchymal cells and immune cells. Future research is necessary to untangle the exact interplay between cancer cells and the microenvironment. Based on these insights, identification of stem cell markers alone for targeted therapy might be less efficient, but addition of therapy that targets important microenvironmental components might lead to improved future treatments.
